# Human Long Noncoding RNA Interactome: Detection, Characterization and Function

**DOI:** 10.3390/ijms21031027

**Published:** 2020-02-04

**Authors:** Marek Kazimierczyk, Marta K. Kasprowicz, Marta E. Kasprzyk, Jan Wrzesinski

**Affiliations:** Institute of Bioorganic Chemistry, Polish Academy of Sciences, Noskowskiego 12/14, 61-704 Poznań, Polandmkasprowicz@ibch.poznan.pl (M.K.K.); martha.kasprzyk@gmail.com (M.E.K.)

**Keywords:** long noncoding RNA, lncRNA, lncRNA interactome, detection lncRNA interactome, lncRNA interactome function

## Abstract

The application of a new generation of sequencing techniques has revealed that most of the genome has already been transcribed. However, only a small part of the genome codes proteins. The rest of the genome “dark matter” belongs to divergent groups of non-coding RNA (ncRNA), that is not translated into proteins. There are two groups of ncRNAs, which include small and long non-coding RNAs (sncRNA and lncRNA respectively). Over the last decade, there has been an increased interest in lncRNAs and their interaction with cellular components. In this review, we presented the newest information about the human lncRNA interactome. The term lncRNA interactome refers to cellular biomolecules, such as nucleic acids, proteins, and peptides that interact with lncRNA. The lncRNA interactome was characterized in the last decade, however, understanding what role the biomolecules associated with lncRNA play and the nature of these interactions will allow us to better understand lncRNA’s biological functions in the cell. We also describe a set of methods currently used for the detection of lncRNA interactome components and the analysis of their interactions. We think that such a holistic and integrated analysis of the lncRNA interactome will help to better understand its potential role in the development of organisms and cancers.

## 1. Introduction

It was estimated from the data obtained during the ENCODE project that 70–80% of the human genome has been transcribed [1], but that only 2% of the genome codes proteins [2]. The function of the genome “dark matter”, non-coding RNAs (ncRNA), has not yet been fully recognized [3]. During the last few years, the application of RNA-seq methods has made it possible to display a divergent repertoire of ncRNAs. ncRNA is classified based on the length of the transcripts [4]. Transcripts containing over 200 nucleotides are considered to be lncRNAs and RNAs smaller than 200 nucleotides were arbitrarily classified as sncRNAs, which encompass miRNA, siRNA, piRNA and tRF [5].

Micro RNAs (miRNAs) are small RNAs made up of 21 to 23 nucleotides with well-defined biogenesis and maturation pathways [6]. They play a pivotal role in controlling gene expression [7,8]. siRNAs perform a similar function, although they originate from long precursors, i.e., repetitive and transposon sequences of the genome [9]. piRNAs (Piwi-interacting RNAs) are the largest group of sncRNAs and are mainly present in germ cells [10,11,12]. The mechanisms of piRNA biogenesis involve several Piwi proteins, resulting in 30–35 nt single-stranded RNAs. This guards germ cells against transposon activity [12]. Moreover, abnormal expression of Piwi proteins and piRNAs results in the lack of fertility in males [10,11,12]. The newly discovered group of snRNA, tRF RNAs, are essentially tRNA fragments [13]. The production of some tRFs under stress conditions is involved in translation repression [13,14].

Much less is known about the biology of lncRNA, which constitutes a significant part of the non-coding genome [4,15]. Recent results achieved using high-through sequencing technologies indicated a high level of diversity in lncRNA classes [16]. They are very heterogeneous in terms of size as lncRNAs number from several hundred to several thousand nucleotides. Furthermore, lncRNA transcripts are derived from different parts of the genome, and lncRNAs are localized in the nucleus or in the cytoplasm. A good example of this is the well-defined nuclear Xist [17] and cytoplasmic Uchl1 lncRNAs [18].

Usually, lncRNAs are transcribed by RNA polymerase II, and like mRNA, are 5′-capped, often spliced and polyadenylated [19]. Generally, compared to mRNA, IncRNAs display modest sequence conservation [20,21]. The reason for this may be that lncRNAs are free of codon preservation constraints [21]. Therefore, the sequence of lncRNAs appears to be less important than their secondary/tertiary structure, which plays a pivotal role [20,21].

**Figure 1:**
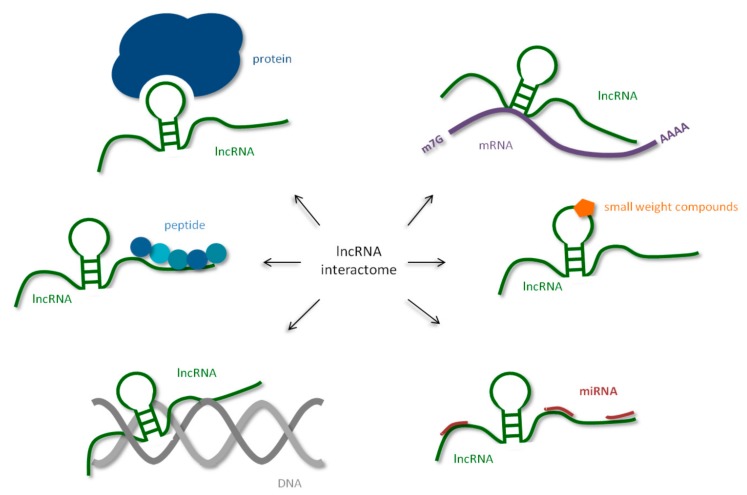
Human long non-coding RNAs (lncRNA) interactome, interaction of human lncRNA with cellular biomolecules.

In this paper, we present recently discovered information about the lncRNA interactome i.e., the cellular components that interact with lncRNA. We describe the interaction of the biomolecules, such as nucleic acids, sncRNA (miRNA), mRNA and DNA with lncRNA. A significant part of the review concerns the importance of lncRNA-protein complexes. Recently, a part of the lncRNA interactome has been found to be made out of short peptides, encoded inside lncRNA, and even small molecules. Their possible impact on lncRNA functions will be discussed. Recently, a part of the lncRNA interactome has been found to be made out of short peptides, encoded inside lncRNA, and even small molecules (Figure 1).

lncRNAs have been discovered to be involved in divergent functions in the human organism, *inter alia*, during cell development and differentiation [15,22,23]. In addition, it appears that the mutation and dysregulation of many lncRNAs may be connected to serious and complex human diseases (Table 1).

Up to 70–80% of the human genome has been transcribed. However, only about 2% of the genome includes protein-coding genes (mRNA) [1,2]. ncRNAs account for the majority of the genome transcripts. The term ncRNA is usually used to refer to RNA that does not encode proteins, however this does not mean that such RNAs do not carry any information or have any functions [16,33,34]. ncRNAs are divided into housekeeping RNAs and regulatory RNAs, based on their functions. Housekeeping noncoding RNAs, including transfer RNAs (tRNAs), small nuclear RNAs (snRNAs), small nucleolar RNAs (snoRNAs) and ribosomal RNAs (rRNAs), are commonly expressed in a constitutive manner. Regulatory RNAs are ncRNAs with a strong regulatory impact on the expression of protein-coding genes. Based on their size, regulatory RNAs can be divided into two groups: sncRNAs (<200 nt) and lncRNAs (>200 nt). Small noncoding RNAs (sncRNAs) are a group which encompasses microRNAs (miRNAs), small interfering RNAs (siRNAs), Piwi associated RNAs (piRNAs), tRNA-derived fragments (tRFs) and circular RNAs (circRNAs). The second group, lncRNAs, which are larger than 200 nucleotides, as has been mentioned previously, do not have the ability to code protein.

Most lncRNAs have been found to be synthesized by RNA polymerase II under the control of the transcriptional activators of the SWI/SNF complex. However, some lncRNAs are transcribed by RNA polymerase III [19,35]. As has been already mentioned, the transcripts are capped, spliced and polyadenylated [36,37].

## 2. The LncRNA Interactome: The Nucleic Acid Story

### 2.1. Interactions of LncRNA with miRNAs, the ceRNA Hypothesis

Despite over two decades of research, the role of non-coding RNAs in human development still remains a mystery. Most of the information available concerns the regulation of the activity of selected genes. There is a lot of evidence that suggests miRNAs contribute to this process, binding to specific mRNA 3’ UTR regions and regulating the expression of these genes [7]. In 2011, Salmena et al. proposed a competitive, endogenous RNA (ceRNA) hypothesis [38], which is supported by a significant amount of experimental evidence [39,40,41]. According to this hypothesis, ncRNAs and miRNAs influence each other. When it comes to the mRNA pool, transcribed pseudogenes, lncRNA, circRNA and other RNAs, there is competition for the same pool of miRNA. When miRNA binds to mRNA, a “seed sequence” containing 2–8 nucleotides, which ensures efficient miRNA interaction, also referred to as the miRNA responsive element (MRE), is required [7,8,42]. It is already known that each mRNA may contain multiple MREs, and thus can be regulated by a number of miRNAs, while one miRNA can potentially regulate dozens of mRNAs. Many experimental findings support the idea that multiple ncRNAs, including sncRNAs, lncRNAs, and circRNAs, as well as pseudogenes, can act as so-called miRNA “sponges”. By sharing identical MREs and competing for common miRNAs, they change miRNA’s activity, which results in modified mRNA translation [41,43].

ceRNAs regulate each other through interactions with shared miRNAs, creating a large-scale regulatory network across the transcriptome, significantly expanding the functional genetic information in the genome (Table 2). In addition, the ceRNAs play an essential role in many biological processes, which is why the destruction of the balance between ceRNAs and miRNAs functions as a regulator. The lack of this balance plays a significant part in disease development and is found in many types of cancer [44].

One important tool used to confirm miRNA–ceRNA interactions is the in silico analysis of the MREs shared by mRNA and ceRNA, such as lncRNA or circRNA. Several computer analysis approaches, such as MARIO, PARIS, LIGR etc, have already been successfully used in the past for performing such studies [75,76,77].

ncRNAs interact with each other during the biological processes that take place in the cell. In order to determine the interaction framework of different RNA molecules, and to determine which RNA molecules can anneal or hybridize to each other in the cell, the potential RNA–RNA interactions must be verified experimentally, in vivo. To achieve this goal, high-throughput sequencing of RNA, isolated by crosslinking and immunoprecipitation (HITS-CLIP) and the application of photoactivatable ribonucleoside enhanced crosslinking, as well as immunoprecipitation (PAR-CLIP) methods, were used [65,78,79]. PAR-CLIP, which is a modification of the HITS-CLIP method, utilizes the UV radiation of cell cultures, in the presence of psoralen derivatives [53,76,79]. These compounds specifically react with RNA, but not with proteins, and generate inter-strand cross-links between RNA’s uridine bases. It has been shown that the integration of these crosslinking methods significantly enhances (over 20-fold) the search efficiency in terms of RNAs interacting with ceRNAs in liver cells, in comparison to *in silico* studies alone [80]. A more precise insight into miRNA–lncRNA interactions has recently been gained through the use of a combination of microarrays and the NGS method [81]. Using microarrays makes it possible for the expression of many sncRNA and lncRNAs to be analyzed in a single experiment. However, more information can be obtained through the analysis of NGS data. Although using both of these methods together is more informative, their high cost can be prohibitive.

The question is whether the ceRNA hypothesis can explain ncRNA’s role in the progression of cancer. It has been calculated that more than 30% of miRNAs are involved in cancer regulation [82,83,84]. Evidence demonstrates that lncRNAs may acts as miRNAs decoy molecules, and may regulate its activity influencing cellular processes, including those that are associated with cancer [39,85]. Comprehensive analyses of ceRNA networks involving lncRNA-associated miRNA have been conducted for many diseases, such as ovarian and prostate cancer, glioblastoma, thyroid carcinoma, as well as breast, lung, kidney and gut cancers [39,86,87]. Usually, lncRNAs were aberrantly expressed and significantly correlated with the cancer prognosis. Recently, it has been shown that the MALAT1 lncRNA can sponge miR-211 as a ceRNA, and potentially up-regulate the PHF19 protein, a component of the polycomb complex, thus facilitating the progression of ovarian cancer [88]. Another HOTTIP lncRNA sponges miR-216a-5p, promoting prostate cancer cell proliferation, migration and invasion [89].

Although there are well-documented examples confirming the ceRNA hypothesis, some scientists find it controversial due to the fact that the expression of an additional mir122 target did not affect its abundance in the hepatocytes and liver [90]. In addition, this observation was confirmed for other ceRNAs; blocking the miRNA binding using antimir oligonucleotides had no physiological influence on miRNA function [91].

### 2.2. Pairing LncRNAs with Messenger RNAs

However, there is another mechanism for regulating RNA activity through its direct base pairing with lncRNA (Table 3). LncRNA and mRNA and the pre-mRNA complementary hybrid may participate in regulating translation by affecting mRNA splicing and editing as well as its stability [92,93].

Most of the information concerning lncRNA–mRNA interactions come from bioinformatics analysis software such as: RNAplexn [99] and LncTar [100]. However, lncRNA–mRNA interactions need experimental verification *via* RNA probing or the aforementioned RNA-RNA crosslinking.

It has been predicted that lncRNA–pre-mRNA interactions may play an important role in alternative splicing. Since almost 90% of human genes are spliced alternatively, controlling this process is important for the development of organisms [92]. lncRNA may effectively regulate splicing; indeed, computer analyses indicate that out of about 24,500 genes, some 21,000 may be affected by the formation of lncRNA–mRNA duplexes. LncRNA may affect pre-mRNA splicing in two ways: by blocking spliceosome assembly involving intron-exon junction or by becoming the target for splicing factors [92]. For example, it has been suggested that MALAT1 lncRNA regulates the alternative splicing of pre-mRNA by controlling the functional levels of transcription factors [94].

The lncRNA-mRNA duplex is a good substrate for RNA adenosine deaminase, a double stranded specific enzyme, which converts adenine to inosine [101]. Inosine differs from adenine in that it possesses a carbonyl group instead of an amino group at position 6 of the purine ring. Such an RNA modification influences the base pairing. Adenine forms a base pair with uracil, unlike inosine, which pairs with cytosine. In the case of lncRNA A to I editing, most of the information comes from bioinformatics analyses [102]. It has been suggested that almost 200,000 editing sites occur in human IncRNAs, and the majority of them (65%) are located within the sites, which significantly changes their secondary structure. Editing may alter the target sites of the lncRNAs and, therefore, the edited and non-edited transcripts may differ in terms of their functions [95,101].

Additionally, lncRNAs influence mRNA stability. Recently Cao et al. reported that lncRNA-assisted stabilization of transcripts (LAST) binds to the 5’ UTR region of *CCND1*mRNA and protects it against possible nuclease targeting in cultured cells [94]. It appears that LAST interacts with other mRNAs. The overexpression of LAST lncRNA has also been observed in several cancer tissues.

### 2.3. LncRNA–DNA Interactions

Several mechanisms of lncRNA recruitment to genomic targets in lncRNA-DNA interactions have been proposed [103] (Table 4). One of them is the direct binding to DNA and the formation of a triple helix. The nature of the RNA-DNA-DNA triple helix formation is described in references [104,105]. Triplexes are formed by weaker, non-Watson-Crick base-pairs, Hoogsteen hydrogen bonds or reverse Hoogsteen hydrogen bonds between the Watson-Crick base-paired dsDNA and the third strand made up of RNA. Triplexes can be either parallel or antiparallel, based on the orientation of the third strand. The orientation of the third strand might be important for its functionality [106,107]. A couple of useful computer tools, which can be used to predict RNA-dsDNA triplex formation and lncRNA–DNA binding sites, such as GRIDseq [108], Triplexator [109], or LongTarget [110] are available. Several techniques, which can be used to search for lncRNA - DNA binding sites, such as Chromatin Isolation by RNA Purification (ChIRP), Chromatin Oligo Affinity Precipitation (ChOP), Capture Hybridisation Analysis of RNA Target (CHART) and RNA Antisense Purification (RAP), have been reviewed by Chu et al. [111] and Vance and Ponting [112].

There are several examples where lncRNA can form a triple helix with dsDNA, such as FENDRR, DHFR, Khps, PARTICLE or NEG3. FENDRR lncRNA forms a triplex structure with gene (*Foxf1, Pitx2*) promoters, creating binding sites for the polycomb repressive complex (PRC2) and regulating the expression of its target genes [114,115]. FENDRR plays an important role in carcinogenesis. The decrease of this lncRNA is associated with gastric cancer. LncRNA DHFR has been shown to inhibit the transcription of *Dfhr* mRNA by creating a triplex structure with the DHFR promoter [122,123]. This interaction results in lncRNA DHFR binding to the TFIIB transcription factor, preventing the formation of a transcription initiation complex. The human heart tissue-specific lncRNA, Khps1, interacts with a homopurine stretch, upstream of the promoter of sphingosine kinase SPHK1, and the recruitment of p300/CBP (histone acetyltransferase) [113]. p300/CBP changes chromatin’s state to active, which allows transcription factor E2F1 to bind and enhance SPHK1 expression. Promoter of MAT2A-antisense radiation-induced circulating lncRNA (PARTICLE) is expressed in response to low-dose irradiation [127]. It has been shown to form a triplex upstream of the methionine adenosyltransferase promoter (MAT2A). PARTICLE leads to the methylation of MAT2A by G9a and PRC2 complexes, which represses transcription. MEG3 binding sites have been shown to contain higher levels of GA-rich sequences. These sites help to guide MEG3 to its targets, by forming RNA-DNA triplexes [117]. Moreover, MEG3 regulates the activity of the TGF-β genes cooperating with the PRC2 complex. AIR lncRNA, an antisense promoter transcript located in intron 2 of the insulin-like growth-factor type-2 receptor gene, has been found to occupy gene *Slc22a3* promoter of cation transporter and recruit H3K9 histone methyltransferase G9a to epigenetically repress transcription [113]. Numerous genomic binding sites, such as NEAT1 and MALAT1, have also been identified for lncRNAs [119,120,121]. Most of the sites include active chromatin at highly expressed genes. Telomeric repeat-containing RNAs (TERRA), also known as TelRNAs, has been associated with telomeric chromatin, thus its involvement in telomere shortening has also been proposed [124,125,126]. In vitro experiments revealed that TERRA inhibits telomerase activity and is evolutionary conserved.

## 3. LncRNA–Protein Interactome

By defining the proteins that interact with lncRNA, it is possible to gain some insight into the molecular pathways, in which the lncRNA of interest might be involved [128]. Due to their significant size, reaching up to tens of thousands of nucleotides, lncRNAs have been shown to act as guides, signals, decoys and scaffolds for many different proteins [129,130]. Thus, it is important to detect which proteins form complexes with specific lncRNAs. There are a couple of approaches to try out when looking for lncRNA–protein interactions. The method to be chosen depends, among other things, on whether we focus on RNAs (RNA-centric methods) or proteins (protein-centric methods) [131]. Electrophoretic Mobility Shift Assay [EMSA] was the first method used to analyze RNA–protein interactions [132]. A ^32^P, fluorescent or chemiluminescent labeled lncRNA probe is incubated with cell lysate proteins and analyzed using non-denaturing, polyacrylamide gel electrophoresis [132,133,134]. The so-called pull down assay works in a similar fashion [131,135]. The biotin-labeled RNA is incubated with a cell lysis protein mixture to bind the interacting proteins. Subsequently, the complex is immobilized on streptavidin-agarose, purified, and detected using Western blotting [136]. Currently, there are three methods allowing for the detection of the lncRNA - protein interactome in use. The first method, immunoprecipitation, is usually used to prepare the RNA protein complexes. The second method combines crosslinking and immunoprecipitation (CLIP) [137,138]. This approach makes it possible to identify the proteins bound to the selected lncRNA, and to determine the protein binding sites in the lncRNA sequence. The last of the methods, Next Generation Sequencing (NGS), is used to analyze lncRNA - protein interactions, which significantly speeds up the analysis of RNA-protein complexes. There are several variants of the CLIP method, like HITS-CLIP, PAR-CLIP, iCLIP, etc. HITS-CLIP was developed as a genome-wide tool, designed to map protein-RNA binding sites in vivo [139]. The Photoactivatable-Ribonucleoside-Enhanced Crosslinking and Immunoprecipitation (PAR-CLIP) method uses cells cultured in the presence of nucleotide analogs, such as 4-thiouridine (s^4^U) or 6-thioguanosine (s^6^G). Thio-nucleotide analogs are incorporated into the newly synthesized RNAs. The crosslinking of proteins to modified lncRNAs makes it possible to precisely determine the type of protein and its binding site in the lncRNA sequence [140].

Because sequencing is an inherent part of all of them, it is crucial to pick the right data analysis methods in order to obtain the necessary information. In silico analysis makes the prediction of the structure, function or interaction of lncRNA-protein complex possible through screening the annotated sequences or structural motifs, like RNA-binding sites. There are also many user-friendly lncRNA databases, which collect information about sequences, which have already been annotated, such as lncRNAdb [141], LNCipedia [142], or NONCODE 2018 [143].

Due to the wide range in lncRNA size, which span from several hundred to several thousand nucleotides, there are many different sites proteins may bind to. Indeed, in the cell lncRNAs exist mainly in the form of RNA-protein complexes (Table 5). It has been suggested that RNA molecules are the perfect scaffolds for protein binding [144]. An estimated 5% of lncRNAs may bind to about half of the interacting proteins. As has been shown in Table 3, there are lncRNAs that specifically bind one protein, and also lncRNAs which bind multiple proteins. For example, many proteins interact with HOTAIR lncRNA [34,145,146,147]. However, lncRNA, lnc-DC interacts only with the STAT 3 protein [148].

The results of the analyses presented in Table 3 clearly show that lncRNA-protein complexes regulate many cell processes: transcription and splicing, as well as gene expression. It should be emphasized that many human lncRNAs interact with the polycomb repressive protein complexes (PRC1 and PRC2) [173,174]. Both protein complexes interact with chromatin and are involved in chromatin remodeling as well as the epigenetic regulation of gene expression via DNA and histone modification [175]. A variety of molecular investigations authenticate the association between lncRNAs—such as HOTAIR, Kcnq1, Air and chromatin remodeling complexes, such as PRC1 and PRC2, which mediate ubiquitination and histone methylation, respectively (Table 4). At the *Kcnq1* locus, the lncRNA Kcnq1ot1 interacts with members of the PRC1 and PRC2 complex proteins [149,150]. At the *Igf2r* locus, Air lncRNA associates with the histone methyltransferase, G9a [154]. HOTAIR recruits PRC2 at the *HOXD* locus, to induce the silencing of the target genes [145,147]. Another example is the heterogeneous nuclear ribonucleoprotein complexes (hnRNP), which encompass several RNA binding proteins involved in gene expression, including pre-mRNA processing, mRNA stability, and translation [129,176]. However, a recent analysis of significant amounts of literature, concerning the interactions of PKC2 proteins with lncRNAs, showed that these interactions are either promiscuous or that the methods used to detect them have a lot of noise [177].

The interactions of the hnRNP protein with various lncRNAsplay a significant role in many cell functions [178]. As mentioned in Table 4, hnRNP interacts with LINC–p21 as well as PNKY lncRNA, thus regulating transcription and alternative splicing, respectively [159,160]. In addition, the THRIL lncRNA (TNFα and hnRNPL related immunoregulatory lncRNA) plays a key role in innate immune responses as well as in inflammatory diseases in humans [172]. To summarize, lncRNAs are targets of many proteins, whereas lncRNA-protein complexes perform many functions in the cell by participating in various cellular processes (Table 5).

## 4. Peptides—A New Factor in the LncRNA Interactome?

LncRNAs are RNAs defined as having a size exceeding 200 nucleotides and being a non-coding part of the genome. This means that lncRNAs do not encode proteins, i.e., they do not harbor an open reading frame. However, recent studies have revealed that a subset of lncRNAs that code small peptides, usually shorter than 100 amino acids, exists [179,180,181]. These lnRNAs are localized in the cytosol and contain only a single-exon-sequence coding peptide.

The detection of lncRNA-origin peptides is not easy, because computer analyses predict many Open Reading Frames (ORF), however, only a few of them are actively translated. It has been calculated that about 23% of the transcribed lncRNAs have been translated [182]. Moreover, the expression of many peptides is weak, and they are difficult to detect, especially in the case of peptides that lack sequence homologies to known proteins. Many methods for detecting peptide expression combine bioinformatic algorithms and experimental verification. An excellent overview of bioinformatics tools used to search for potential ORF in lncRNAs was put forth by Choi et al. [183]. The simplest way of verifying the predicted ORF involves in vitro translation methods. Constructing a template and performing translation with the use of rabbit reticulocyte, with ^35^S methionine and SDS electrophoresis, makes it possible to determine the size of the predicted peptide [184]. This is important, because due to its size (>200 nucleotides), lncRNA contains many AUG start codons and the encoded peptides vary in length.

The identification of peptides in protein samples isolated from tissues or culture cells, is possible by applying the Western blot method with the appropriate antibodies, following peptide purification. Moreover, the development of mass spectrometry techniques has made this method perfect for identifying such peptides [185]. Another approach involves the use of the ribosome profiling, in connection with NGS methods [186]. In this approach, the RNAs bound to polysomes are purified using sucrose gradient centrifugation, and then, after nuclease digestion, the RNA fragment protected by ribosomes is recovered. These RNA fragments are sources for library preparation and sequencing and the use of bioinformatics tools in genome mapping, which makes the identification of the ORF region possible.

The fundamental issue associated with these bifunctional RNAs is the determination of the role of lncRNA and its coding peptide. The initial question is whether they act as a complex or as separate compounds. The next question is whether the maternal lncRNA, which contains ORF, interacts with its own peptide.

Analysis of available data indicates that the size of the peptide is not connected with the size of the maternal lncRNAs. An 8.7 kb long MALAT1 lncRNA codes a peptide containing 213 amino acids, while the four times shorter XGAT1 lncRNA [2.1 kb] codes a peptide containing 210 amino acids, which is identical in size. [183,187]. Dissection impact on the cell selected lncRNA and translated from its ORF peptide is difficult. Recently, the role some peptides play in cell functions has been explained. The 46 amino acid peptide, myoregulin, which is encoded by a 16.5 kb long skeletal, muscle-specific lncRNA, LINC00948, regulates the regeneration of skeletal muscles by interacting with sarcoplasmic reticulum Ca^2+^-ATPase (SERCA) [188], lysosomal v-ATPase [189] or removing SERCA inhibitors [190].

Moreover, only the HOXB-AS3 peptide, not lncRNA, is critical for suppressing colon cancer growth, by blocking pyruvate kinase M [PKM] splicing, miR-18a processing, and the subsequent glucose metabolism reprogramming [191]. On the other hand, *HOXB-AS3* lncRNA could also regulate the cell cycle progression of OCI-AML3 cells in Npm1 mutated acute myeloid leukemia. Observations suggest that there might be an unknown interaction between the lncRNA and peptides, however, this hypothesis requires further investigation.

## 5. Do Small Compounds Influence LncRNA Activity?

Many RNAs form constrained structures containing “pockets” that bind small molecular weight compounds. This repertoire of RNAs includes long RNAs and small RNAs, such as rRNA and mRNA, as well as ribozymes, riboswitches, and aptamers, which were discovered during the last twenty years [192,193]. The question is whether chemical compounds with a low molecular weight, which regulate lncRNA activity by structure-specific binding, really exist.

The recently determined 3.1 Å resolution MALAT1 crystal structure revealed a constrained structure, containing a bipartite triple helix at the 3′ end [194]. The disruption of the stability of the helix by a point mutation resulted in MALAT1 accumulation in the cell [195]. The recognition of the triple helix by a methyltransferase enzyme strongly supported its functional role [196]. Small molecules, which destabilize this MALAT1 structural element, make it possible to regulate lncRNA functions [197]. XIST is one of the best-characterized lncRNAs. It functions as the major effector of the X chromosome inactivation (XCI) process in mammals. Specific structural domains, namely six tandem hairpin repeats (A–F), are crucial to its functioning in the XCI process. Structural insight into the A and F repeat region of the hairpins revealed an intricate architecture within specific functional modules [198]. In cellulo chemical probing of the entire 18k.b. transcript enabled the discovery of an additional domain at the 3’ end, connected with XIST localization [199]. Decoding the structure should make it possible to identify small molecules, which have the ability to recognize lncRNA’s structural elements. It will also elucidate the role they play in development and disease.

Research on the lncRNA structure using low molecular weight compounds (DMS, DEPC, Pb2+ ions, etc) has shown it is conserved [200]. This means that structural elements, such as loops, bulges and base-paired regions, which occur in small RNAs, are also found numerous times in lncRNA and may be places where various small molecules bind. Information concerning the interactions of small compounds with lncRNA is still limited. Fatemi et al. identified small molecules, which bind to the lncRNA-protein complex, using high-throughput compound screening methods, i.e., the Amplified Luminescent Proximity Homogeneous Assay [201]. They reported on the specific and quantifiable binding of the brain-derived neurotrophic factor antisense lncRNA to a component of the PCR2 complex, protein EZH2, and also identified a small-molecule inhibitor–ellipticine, that upregulates its downstream target genes. It was also reported that telRNAs, which form the G-quadruplex, target alkaloid quindoline derivatives [202]. The binding of this compound inhibits proliferation and causes G2/M phase arrest in osteosarcoma cancer cells as well as induces DNA damage response and apoptosis. Recently, it has been shown by Shi et al. [203] that the binding of fluorescent peptide derivative NP-C86 to GAS5 lncRNA stabilizes its structure.

## 6. Concluding Remarks and Perspectives

Although 70–80% of the genome has been transcribed, it has been discovered that only 2% of the genome encodes protein sequences. 80% of the remaining ncRNA pool is made up of lncRNA. As this review shows, lncRNAs are not just cellular junk but are involved in many processes such as transcription, translational regulation or in cell development in general. Long noncoding RNA has been shown to interact with a range of cell biomolecules such as other RNAs (miRNAs, mRNAs) and DNA to form the lncRNA interactome, which is involved in life processes. A significant part of the lncRNA interactome is associated with the formation of complexes with proteins or even, as has been recently discovered, peptides. Given the size of the entire lncRNA interactome, the examples that have been studied to date are just the tip of the iceberg. The fact that interference disorders in the lncRNA interactome are the cause of various pathogenesis pathways, including cancer, neurodegenerative, and immunological diseases, is also an impetus for further research.

In the future, one can expect new lncRNA annotation tools will be developed, such as new DNA and RNA sequencing methods (nanopore sequencing) and new bioinformatics methods [204,205]. The development of new methods of analysis, like mass spectroscopy makes it possible to detect biomolecules that bind to lncRNA, including proteins, peptides and low-molecular compounds. It is also important to determine how the lncRNA interactome influences cellular processes, as well as the impact it has on various stages of the organism’s development. New DNA editing methods (CRISP/Cas9 and RNA (CRISP/Cas13 [206,207] could be helpful in achieving these goals. The destruction of genes encoding proteins or lncRNA and miRNA seems to be a powerful method of testing the lncRNA interactome. It seems particularly important to determine the role of lncRNA in the pathogenesis of various diseases and as a biomarker of the disease state, for example, during the cancerogenesis processes. However, modulating lncRNA activity with low-molecular compounds (alkaloids, antibiotics, peptides, etc.) may be used for therapeutic purposes in the future.

To summarize, the lncRNA interactome is a large group of biomolecules, related to and interacting with lncRNA, that play a variety of roles in cell development and the pathogenesis of various diseases.

## Figures and Tables

**Table 1:** Examples of pathogenesis related to lncRNA dysregulation.

Disease	LncRNA	Impact on Pathogenesis	Mechanism
Colon cancer	DACOR1 [24,25]	Downregulated in colon tumors	Interacts with and inhibits DNA methyltransferase DNMT1
Lung cancer	HOTAIR [26]	Impacts proliferation, survival, invasion, metastasis, and drug resistance in lung cancer cells	HOTAIR may promote dedifferentiation of lung epithelial cells through two distinct mechanisms, i.e., transcriptional repression of *Hox5* and ubiquitin-mediated proteolysis of Ataxin-
Prostate cancer	LINCRNA-p21 [27]	Decreases prostate cancer cell proliferation	LINCRNA-p21 inhibits many genes expression in a p53-dependent transcriptional response
Parkinson’s disease	H19, LincRNA-p21, MALAT1, and SNHG1 [28]	H19 is significantly downregulated in Parkinson’s disease while LincRNA-p21, Malat1 and SNHG1, are significantly upregulated.	Associated with synaptogenesis, proliferation, apoptosis, precedes Parkinson’s disease
Leukemia	MALAT1 [29]	Inhibiting multiple myeloma growth	Involved in multiple myeloma DNA repair and cell death.
Cardiovascular diseases	GAS5 [30]	Promotes the development and progression of myocardial infarctions	Targeting of the miR-525-5p/CALM2 axis
Diabetes	HI-LNC901, PLUTO [31]	Implicated in pancreatic islet function	Regulates the transcription of *PDX1* gene of pancreatic β cell
AIDS	LINC00173 [32]	Regulates cytokines in T cells	Presumably involved in transcriptional regulation

**Table 2:** Selected lncRNAs interacting with microRNA (miRNA) and their functions.

	LncRNA	Interacting miRNA	Function in the Cell
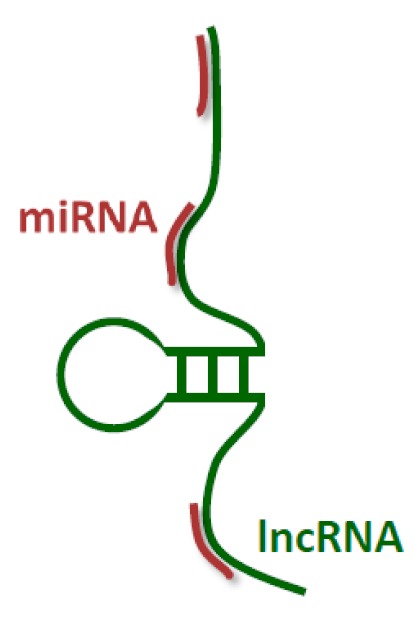	LINC-ROR	miR-138, miR-145, miR-204 [45,46]	competing endogenous RNAs
	LNCRNA-KRTAP5-AS1, LNCRNA-TUBB2A	specific miRNA [47]	
	CASC2	miR-21, miR-18a [48,49]	
	CDR1AS/CIRS-7	miR-671, miR-7 [50,51]	
	LINC-MD1	miR-133, miR-135 [52]	
	MDRL	miR-361 [53]	
	HULC	miR-372 [54]	
	LINC-223	miR-125-5p [55]	
	LNCARSR	mir-34, miR-449 [56]	
	LNCND	miR-143-3p [57]	
	UFC1	miR-34a [58]	cell cycle
	LINC00152	miR-138 [59]	
	MALAT1	miR-101, miR-217, miR-9, miR-125b [60,61,62]	controlling proliferation and senescence
	UCA1	miR-1 [63]	
	BACE1-AS	mir-485-5p [64]	modulation of mRNA stability
	H19	miR-106a, miR-17-5p, miR-20b, let-7 [65,66]	transcriptional regulation
	HOTAIR	miR-34a, miRNA-141, miR-130a, miRNA-let7 [67,68,69]	
	MEG3	miRNA-29 [70]	
	GAS5	miR-21 [71]	
	HOST2	let-7b [72]	
	PCAT-1	miR-3667-3p [73]	post-transcriptional regulation
	LINCRNA-P21	let-7 [74]	modulation of translation

**Table 3:** Some examples of lncRNA–mRNA pairing.

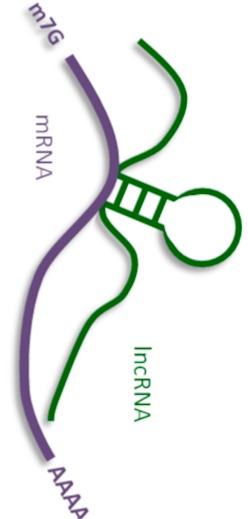	**LncRNA**	**mRNA**	**Impact on**
	SAF	FAS [92]	alternative splicing
	ZEB NAT	ZEB [92,93]	
	MALAT	CAMK2B, CDK7, SAT1, HMG2L1, ARHGEF1, B- MYB [94]	
	PCA3	PRUNE [95]	A–I mRNA editing
	LAST	CCDI [96]	mRNA stability
	BACE1	BACE1 [97]	
	LINCRNA p21	JUNB, CTNNB1 [98]	

**Table 4:** Examples of lncRNA interacting with DNA and their postulated function.

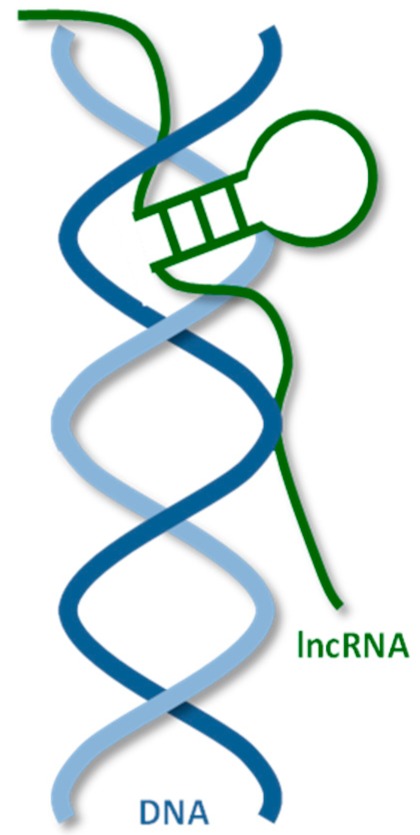	**LncRNA**	**Interacting DNA Region**	**Function**
	AIR	Slc22a3 promoter [113]	Epigenetic control of gene expression
	FENDRR	Foxf1, Pitx2 promoters [114,115]	
	TUNA	Nanog, Sox2 and Fgf4 promoters [116]	
	MEG3	TGF-β pathway genes [117]	
	PARTICLE	Upstream of MATSA promoter [118]	
	NEAT1	Multiple binding sites [119,120]	Paraspecle formation
	MALAT1	Multiple binding sites [121]	Alternative splicing regulation, promotes metastasis
	LncRNA DHFR	DHFR promoter [122,123]	Transcription regulation
	TERRA	Telomers [124,125,126]	Telomer replication control

**Table 5:** lncRNA-protein complexes involved in the regulation of cellular functions.

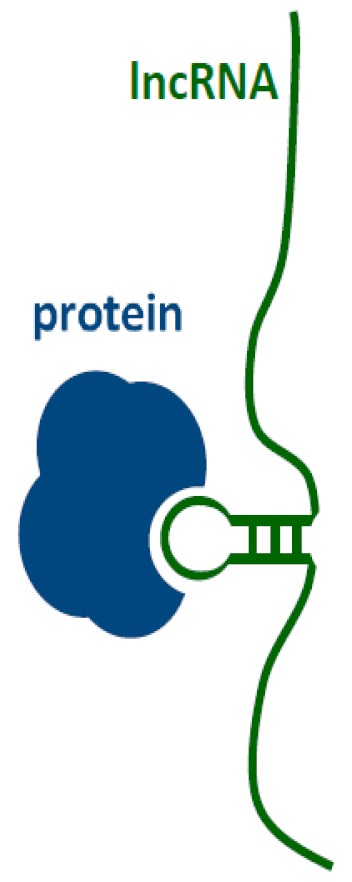	**LncRNA**	**LncRNA Length**	**Interacting Protein(s)**	**Function**
	KCNP1ot1	91.7 kb	PRC1, PRC2, G9a [149,150]	Epigenetic control of gene expression
	XIST	19 kb	PRC2, hnRNP U, YY1 [151,152]	
	AIR	4.3 kb	G9a [153,154]	
	ANRIL	3.8 kb	PRC1 [155]	
	HOTTIP	3.8 kb	WDR5, MLL [156]	
	HOTAIR	2.4 kb	PRC2, LSD1/CoREST/REST [145,146,147]	
	H19	2.3 kb	PRC2 [157]	
	DEANR1	4.9 kb	SMAD2/3 [158]	Transcription regulation
	LINCRNA-p21	2.7 kb	HnRNP [159,160]	
	PANDA	1.5 kb	SAFA [161,162]	
	GAS5	1.0 kp	GR (NR3C1) [163]	
	LNC-DC	630 bp	STAT-3 [148]	
	7SK	330 bp	P-TEFb [164,165]	
	MIAT (Gomafu)	10 kb	SF1 [166]	Alternative splicing
	MALAT (NEAT2)	8.7 kb	SR [167]	
	PNKY	1.6 kb	hnRNP [168,169,170]	
	BC1	152 bp	EIF4A/eIF4B, PABP [171]	Translation regulation
	THRIL	2.9 kb	HnRNP [172]	Immune response
